# Medical students’ attitudes toward gay men

**DOI:** 10.1186/1472-6920-12-71

**Published:** 2012-08-08

**Authors:** Kabir Matharu, Richard L Kravitz, Graham T McMahon, Machelle D Wilson, Faith T Fitzgerald

**Affiliations:** 1 School of Medicine, University of California, Davis, Sacramento, CA, 95817, USA; 2 Division of General Internal Medicine, University of California, Davis, Sacramento, CA, 95817, USA; 3 Center for Healthcare Policy and Research, University of California, Davis, Sacramento, CA, 95817, USA; 4 Department of Medicine, Brigham and Women’s Hospital, Boston, MA, 02115, USA; 5 Division of Biostatistics, University of California, Davis, CA, 95616, USA

**Keywords:** Homosexuality, Medical students, Bias

## Abstract

**Background:**

Healthcare providers’ attitudes toward sexual minorities influence patient comfort and outcomes. This study characterized medical student attitudes toward gay men, focusing on behavior, personhood, gay civil rights, and male toughness.

**Methods:**

A cross-sectional web-based anonymous survey was sent to medical students enrolled at the University of California, Davis (N = 371) with a response rate of 68%.

**Results:**

Few respondents expressed negative attitudes toward gay men or would deny them civil rights. More negative responses were seen with respect to aspects of intimate behavior and homosexuality as a natural form of sexual expression*.* Men and students younger than 25 years old were more likely to endorse negative attitudes toward behavior as well as more traditional views on male toughness.

**Conclusions:**

We show that an important minority of students express discomfort with the behavior of gay men and hold to a narrow construction of male identity. These findings suggest that competency training must move beyond conceptual discussions and address attitudes toward behaviors through new pedagogical approaches.

## Background

Though medical care in the United States has promoted health and longevity, many disparities persist even after socioeconomic status and other factors have been accounted for [1,2]. Sexual minorities are a group for whom disparities in health are prevalent and problematic [3,4]. Lesbian, Gay, Bisexual, and Transgender (LGBT) individuals are often “invisible” due to the ability of members to hide their status and thus avoid bias because they may be construed as “different” from their peers [5,6]. As a result of familial, societal and religious pressures, many LGBT individuals hide their status and relatively few make their orientation known to their health care providers. Those who disclose their orientation may find that their physician is unprepared and even unwilling to discuss same-sex relationships and behavior [7].

Medical educators recognize the need for physicians to understand and interact with patients of different cultural backgrounds since prejudices of medical students and physicians have demonstrable and important effects on patient wellness [8]. Often, health care providers are not aware of their biases, which can remain unconscious. This leads to deleterious patient outcomes due to assumptions that one is heterosexual and negative caregiver attitudes that are not openly discussed in medical education settings [9]. Social desirability bias often limits the disclosure of negative attitudes [10,11]. Health care providers who have negative attitudes toward same-sex behavior have been found to provide inadequate care for LGBT individuals [12]. Furthermore, studies assessing prejudicial reactions of health care providers have been limited in recent years. In 1982, a questionnaire sent to members of the San Diego County Medical Society revealed that 23% of respondents exhibited prejudiced attitudes and 30% would reject a highly qualified gay applicant to medical school [13]. Another study revealed “low-grade homophobia” among medical students that did not change significantly despite panel discussions and clinical experiences [14]. A more recent look at San Diego County Medical Society members’ view on gay students revealed a much decreased prevalence of sexual prejudice, but one that still existed and was associated with phobias about human immunodeficiency virus infection [15].

The objectives of this study were to determine medical students’ attitudes toward gay male behavior, persons, civil rights, and male toughness. We chose to focus on gay men in the context of “masculinity” or “normative” views on male gender roles because there are no studies to date suggesting that these concepts should be discussed in medical school lectures. We hypothesized that individuals would not exhibit prejudice toward a person’s identity, but would exhibit bias when reflecting on the intimate practices of others. We sought to investigate the prevalence and correlates of negative attitudes toward gay identity and behavior. We predicted negative attitudes toward gay men among older students and males based on public surveys suggesting these populations have more aversive reactions [16].

## Method

### Setting

The University of California, Davis, School of Medicine has a diverse student enrollment comprising 25% underrepresented minority students. Approximately 2.3% of students across all years self-identify as LGBT (personal communication, Office of Diversity). There is an active LGBT students’ organization within the campus. At the time of this writing, the University of California, Davis School of Medicine devotes 10 hours of curricular time to LGBT issues during the course of four years’ training, which is above the mean hours (5) spent nationally. A survey of medical school deans demonstrated that 44% of schools provide “fair” instruction in LGBT issues [17]. The medical school curriculum also emphasizes the instruction of culturally sensitive care through a three-year “Doctoring” curriculum in which students are exposed to simulated patients from different ethnic and sexual backgrounds. Role-played interactions trigger feedback and discussion in small groups. In addition, faculty members provide formal lectures and experiential learning on caring for diverse populations.

### Survey generation and scoring

We developed a 20-item survey incorporating items from previously validated instruments to assess attitudes toward gay behavior, persons, and civil rights [18-21]. In addition, a scale measuring male toughness was included because normative attitudes on this dimension have been associated in prior studies with negative attitudes towards gay men [22]. Item selection was refined iteratively to generate the shortest comprehensive instrument. Each item on the four subscales was rated on a seven point Likert scale from strongly disagree to strongly agree. Several items on the scale were reverse scored so higher scores corresponded to more negative reactions. We also recorded the respondents’ sex, age, race/ethnicity, and sexual orientation. Those who identified as LGBT were excluded from the final analysis due to small numbers (*n* = 13).

### Survey implementation

We conducted a cross-sectional survey of currently enrolled medical students from December 2010 to January 2011. The study was approved by the University of California, Davis Institutional Review Board. Online consent included a full description of the study, including potential harm (no more than minimal but with potential for discomfort). Participants received no monetary or non-monetary incentive for their participation in completing the survey and were not required to fill out all questions of the survey. Questionnaires were sent out to an e-mail listserv used by current medical students. The message discussed the survey’s intent to better characterize medical student attitudes toward gay men for the assessment of educational services and development of appropriate modules in the future. Three reminder e-mails were sent to ensure the highest possible number of respondents. A subject was recorded as a non-respondent if he or she did not provide consent after accessing the survey, or failed to fully complete a survey. The questionnaire was password-secured and available within 60 days from the initial announcement.

### Statistical analysis

The four constructs measured from the survey questions were attitudes toward gay behavior (*behavior*), people (*persons*), civil rights (*civil rights*), and male toughness (*toughness*). Constructs were estimated by the sum of the responses to the relevant questions.

The *age* variable was trichotomized: less than 25 years old, between 25 and 28 years old, and greater than 28 years old. The *race/ethnicity* variable similarly had three categories: White, Asian or Other, due to low representation of racial/ethnic groups other than White and Asian. The *sexual orientation* variable also had three options: Heterosexual, Homosexual and Bisexual. Homosexual and bisexual individuals were not included in the final analysis due to their overall low numbers.

A full exploratory and graphical analysis of the scales was conducted using histograms, scatterplots and Cronbach’s alpha to assess distribution and linearity assumptions and scale score reliability, respectively. If the histogram indicated a non-normal distribution then various transformations were attempted to correct the distribution. If no transformation appeared to achieve normality or linearity, non-parametric analyses were used. The Kruskal-Wallis (KW) test of differences in medians was performed to assess differences in each of the four constructs between the different categorical variables: *gender*, *race/ethnicity*, and *age*. Additionally, Spearman correlations were calculated to estimate the relationships between the scales.

There were some missing data due to item non-response. These missing data were imputed by means of chained equations in R® using multinomial regression, and the complete, imputed data were analyzed in SAS as above [23,24]. Imputation of missing item responses is necessary to avoid item non-response bias, a common problem with survey data [25,26]. The imputation was repeated 5 times and each imputed data set analyzed. Analysis results were compared between the 5 imputations and the original, incomplete data. If no large differences were observed between the 5 imputations and their analyses, no correction for multiple imputation variance deflation was performed or presented.

## Results

251 out of 371 medical students responded (response rate: 68%). The histograms revealed a highly skewed, non-normal distribution, thus requiring non-parametric methods to determine significance. The results of the multiple imputations suggested that there were no large differences between any of the analyses, with p-values differing only in the third decimal place. For this reason, the first imputation was used and all results are from this complete data set.

Respondents had an average age of 27 years (range from 21 to 45 years): 94.8% of respondents were heterosexual. Of those who completed the survey, 59.4% of the respondents were women and 50.6% were Caucasian. Additionally, we delineated three age groups to determine whether age, irrespective of year of enrollment, influenced attitudes toward homosexuality. We did not ask respondents about their country of origin nor specific year in the program (Table 1).

**Table 1:** Descriptive statistics of respondents

**Characteristics of Respondents**		***n*(%)**
**Age group (years)**	< 25	52 (21.1)
	25-28	149 (60.6)
	> 28	45 (18.3)
**Gender**	Male	102 (40.6)
	Female	149 (59.4)
**Sexual orientation**	Heterosexual	238 (94.8)
	LGBT^a^	13 (5.2)
**Race/Ethnicity**	White (non-Hispanic)	126 (50.6)
	Asian	61 (24.5)
	Other	62 (24.9)

^a^ Lesbian, Gay, Bisexual, or Transgender (LGBT).

Cronbach’s alpha coefficient showed that survey elements were moderately internally consistent across subscales of gay behavior (.86) and male toughness (.82). However, scale score reliability for persons and civil rights (.53 and .57, respectively) was less robust, perhaps due to the low number of questions in those subsections (Table 2). Nearly all respondents endorsed positive attitudes toward gay persons and their civil rights, though skewed data was obtained for the behavior and male toughness scales.

**Table 2:** Description of attitudes

**Homosexual behavior α = 0.86 (.78-.99)**	***n***	**% Agree/Strongly agree (6/7)**	**% Neutral (5)**	**Mean (Standard deviation)**	**Minimum Observed**	**Maximum observed**
Homosexual behavior between men is morally wrong.	238	5.9	2.0	1.7 (1.5)	1.0	7.0
Male homosexuality is a perversion.	238	4.2	1.0	1.5 (1.3)	1.0	7.0
If a man has homosexual feelings he should overcome them.	238	2.9	0.4	1.4 (1.1)	1.0	7.0
If I saw two men holding hands I would be more disgusted than if I saw a man and a woman holding hands.	238	4.2	6.0	1.8 (1.5)	1.0	7.0
The thought of two men having sex is more disgusting than the thought of a man and woman having sex.	238	16.4	12.0	3.0 (2.1)	1.0	7.0
Male homosexuality is as natural a sexual expression in men as heterosexuality.^*^	238	82.3	6.4	2.8 (2.1)	1.0	7.0
**Homosexual persons α = 0.57 (.29-.84)**						
Gay men are disgusting.	238	.42	0.4	1.3 (0.8)	1.0	7.0
I won’t associate with gay men if I can help it.	238	0.4	0.0	1.1 (0.6)	1.0	7.0
Gay men are a threat to the safety of children.	238	0.0	0.4	1.1 (0.4)	1.0	5.0
I would not mind having male friends who are gay.	238	97.9	0.4	1.3 (1.0)	1.0	7.0
**Civil rights α = 0.59 (.41-.77)**						
Sexual behavior between men should be illegal.	238	0.0	0.0	1.1 (0.4)	1.0	4.0
Gay men should have the same civil rights as anyone else.^*^	238	98.7	0.4	1.2 (0.8)	1.0	7.0
I am happy when I hear about gay men fighting for rights in society.^*^	238	92.0	1.7	1.8 (1.4)	1.0	7.0
**Male toughness α = 0.82 (.61-1.0)**						
When a man is feeling a little pain he should try not to show it.	238	8.0	10.0	2.6 (1.7)	1.0	7.0
Nobody respects a man who frequently talks about his worries, fears, and problems.	238	5.5	12.0	2.6 (1.6)	1.0	7.0
A good motto for a man would be “when the going gets tough, the tough get going.”	238	16.0	10.0	3.6 (1.7)	1.0	7.0
A young man should become physically tough even if he isn’t big.	238	6.3	11.3	3.0 (1.6)	1.0	7.0
Fists are sometimes the only way out of a bad situation.	238	4.2	9.5	2.3 (1.6)	1.0	7.0
A real man enjoys a bit of danger now and then.	238	4.6	10.0	2.6 (1.6)	1.0	7.0
In some situations a man should use his fists even if his wife or girlfriend would object.	238	1.7	5.7	2.0 (1.3)	1.0	7.0

^*^Reverse scored for scale calculation (values of 1, 2, or 3 corresponded to 7, 6, or 5 respectively).

However, for some respondents, the thought of two men holding hands or having sex was more “disgusting” than the thought of a man and a woman engaging in the same acts; a significant minority of students found homosexuality to be an unnatural form of sexual expression. A significant portion of respondents exhibited traditional views on male gender roles with respect to concepts of toughness and aggression (Table 2).

We next sought to determine if respondents from different demographic groups scored differently. The results evidence disparities in response according to respondents’ demographic characteristics (Table 3).

**Table 3:** Average score on scales according to demographic variables

**Variable**			**(Mean, Standard deviation)**		
		**Behavior**	**Persons**	**Civil rights**	**Male toughness**
**Age (years)**	< 25	15.7 (10.1)	4.8 (1.4)	4.4 (2.5)	20.6 (7.6)
	25-28	11.2 (6.7)	4.8 (2.0)	4.0 (2.0)	18.0 (7.9)
	> 29	11.5 (6.0)	5.0 (2.0)	4.1 (2.1)	18.8 (7.6)
	*KW*	35	18	4.5	62
	*p-value*	<0.001	<0.001	0.034	<0.001
**Gender**	Male	15.1 (8.1)	5.3 (2.3)	4.5 (2.6)	23.6 (7.1)
	Female	10.3 (6.7)	4.5 (1.4)	3.8 (1.7)	15.4 (6.5)
	*KW*	9.7	1.8	2.3	4.1
	*p-value*	0.0077	0.42	0.31	0.13
**Race/Ethnicity**	White	12.0 (7.5)	4.8 (1.8)	4.0 (1.9)	18.1 (7.8)
	Asian	12.1 (7.4)	4.7 (1.6)	4.3 (2.6)	19.8 (8.3)
	Other	12.8 (8.2)	4.9 (2.2)	4.1 (2.2)	19.1 (7.4)
	*KW*	0.51	0.13	0.09	1.9
	*p-value*	0.78	0.94	0.96	0.38

### Influence of gender

There were statistically significant differences by gender in all four scales as per the Kruskal-Wallis (KW) test, with men scoring significantly higher on *behavior* and *male toughness* (Table 3).

### Influence of race/ethnicity

There was no evidence of differences between racial/ethnic groups for any of the scales (Table 3).

### Influence of Age

For the three age categories, there was a statistically significant difference in the behavior scale, with the <25 year-old group having a larger mean than the other two groups (Table 3).

### Correlates of more negative reactions

Negative attitudes toward gay male behavior moderately correlated with negative attitudes toward gay persons, civil rights, and male toughness. A negative response on the persons scale was moderately correlated with a negative response on the civil rights scale and weakly correlated with male toughness. Additionally, there was a weak correlation between civil rights and male toughness (Table 4).

**Table 4:** Spearman correlations among attitudinal scales

	**Behavior**	**Persons**	**Civil rights**
**Persons**	.54**		
**Civil rights**	.48**	.43**	
**Male toughness**	.42**	.24**	.26**

** p < .001.

## Discussion

This study provides a comprehensive characterization of attitudes toward gay men endorsed by students at a large U.S. medical school comprising a diverse student body. The majority of respondents were affirming of gay men and same-sex behavior. Overt disgust towards gay men was infrequent. However, most questions in the “behavior” scale had wide standard deviations suggestive of diverse attitudes. Substantial minorities of students expressed disgust to gay male behavior that correlated moderately with negative attitudes regarding civil rights and normative notions of male toughness. The results of this study reveal the need for assessing and revising current methods of or approaches to instruction in the care of sexual minorities.

Good doctor-patient relationships require trust and mutual respect. Clinical heterosexism, the assumption that the patient is heterosexual, interferes with the formation and maintenance of a healthy doctor-patient relationship, and important opportunities to engage a gay patient in healthful behaviors are likely to be missed [27]. Though typically covert, biases such as those reported in this study can lead to subconscious actions. Prejudices have been shown to result in overt antigay behaviors [28]. More worryingly, disgust toward gay men experienced by health care providers and respondents to our survey is particularly powerful [29]. Such visceral responses are likely to undermine quality of care and reinforce stigma.

Homosexuality has been considered to be “a natural difference, like left-handedness.” [30] However, when specifically asked, nearly one-third of medical students responded either negatively or ambivalently to the statement that male homosexuality is as natural a form of expression as heterosexuality. Our study demonstrates that some medical students find gay men and their behavior confusing. Studies have demonstrated the direct relationship between implicit and explicit measures of bias toward gay men that is most apparent among heterosexual men and based upon affective responses. Our study measured explicit bias. By its nature, unconscious bias goes unrecognized by people who see themselves as tolerant or at least hoping to “extract conformity with social norms.” [31] Though individuals may feel “comfortable” with LGBT issues in theory, when they are explicitly presented them, it becomes disconcerting. Educational modules must therefore explicitly endorse the “normality” of homosexuality.

Our study has several strengths. The survey had a high response rate. Despite this, it is possible that those who did not respond are less comfortable with discussion of sexually and prejudice. Our results were consistent with a related study on exposure to LGBT patients conducted among medical students at the New York University School of Medicine [32,33]. Our study sought additional components of beliefs about gay men to more comprehensively evaluate for the presence of bias. Since California is historically politically liberal, it is likely that a similar survey administered in a more conservative part of the country would find a higher prevalence of prejudicial beliefs. Although the University of California, Davis, School of Medicine, is a committed leader in the inclusion of LGBT issues in the curriculum, a lack of acceptance was found among a significant minority of students, thus highlighting the need to help students recognize and understand their own biases for better communicating in a socially- and culturally-appropriate manner with patients from all sexual orientations, races, ethnicities, and so forth. There was a discrepancy between the percent of students that let the school administration know they identified as LGBT (2.3%) and those who identified as such on the survey (5.3%). This underscores the notion that some students are more comfortable with their sexuality while others feel less forthcoming.

Several limitations should be considered when interpreting our findings. The results represent the views of a single medical school student population within the United States to a single stigmatized group. Our study did not evaluate a respondent’s year in medical training to determine whether attitudes changed with increased amount of time in school. Most medical students are in their twenties, making conclusive statements regarding age and negative attitudes more difficult because the range is more narrow. The cross-sectional and correlational analyses preclude a broader generalization of how medical students in the United States and globally frame negative responses to gay men, and does not characterize their perceptions of lesbians, bisexual or transgender people, or intersex persons. Given that many questions used the word “disgust,” it is possible that even higher rates of bias would be seen if less extreme words such as “discomfort” or “unease” were used instead. In this study, we utilized explicit measures of bias. The questions exposed self-reported bias, which may not correspond to privately held beliefs. Some individuals may exhibit negative reactions to those different from themselves, but will not divulge this information when directly asked [34]. Future work can augment this study to assess the relationship to beliefs that are explored through measurement of implicit measures to determine if there are discrepancies among students in higher education, where disguise of bias may be more apparent [35].

Efforts to address prejudices, among other biases that may be covert, are needed to facilitate open discussions and thereby improve care. Previous research has shown that individuals who believe that homosexuality is “congenital” exhibit more positive attitudes toward sexual minorities. Labeling or constructing groups as different in speech, appearance, or socio-cultural background has deleterious effects on the ability to provide sound and culturally-adept treatment [36-38]. Several innovative workshops have been developed that help to address the rich needs of this underserved group beyond sexual practice and pathology but more are needed [39]. This includes tackling concepts of gender roles and confronting patient issues that would otherwise be unspoken.

We show that negative attitudes toward gay behavior were linked to more normative views on male gender roles, or how men are expected to act. Our study advances medical education for gay men by supporting the need for lectures to discuss masculinity due to documented correlations with heterosexism [40]. Changing medical student attitudes cannot be done in isolation and should be a component of a comprehensive organizational approach that involves leadership and faculty role models to shape the culture throughout all the clinical and academic venues encountered by students.

There is considerable evidence that competency in dealing with populations experiencing health disparities require systematic development of a competency-based curriculum throughout all of medical school. Developing a comprehensive four-year curriculum around LGBT health is a critical first step. However, even the presence of LGBT patients and lectures disseminating facts may not adequately address underlying biases. More interactive methods that incorporate diverse fields of study, including anthropology and literature, could be used to invoke empathy. Incorporation of interdisciplinary efforts in current educational modules may attenuate biases seen not only towards sexual minorities, but the underserved from various ethnic and social backgrounds.

## Conclusion

Our study demonstrates that sexual prejudice is more likely to be found in male and younger medical students. Because the overall data is not normative, it is difficult to determine whether there was an overall “positive” or “negative” prejudice. The first finding is surprising given that most polls on homosexuality show a more positive attitude among younger respondents. Students may acquire more positive attitudes toward sexual minorities as exposure to LGBT patients and other life experiences increases through medical school. Though attitudes toward gay behavior are complex, equality in the provision of clinical care mandates improved educational interventions. Health professionals must acknowledge biases they have despite discomfort this may cause. The survey utilized in this study could be used at other medical schools to assess the extent of sexual prejudice against gay men and therefore provide impetus to implement curricular changes in concert with a cultural competence committee. Longitudinal studies with specific interventions should be explored to determine whether prejudice reduction strategies could improve prejudicial reactions using introspection and facilitated clinical encounters. Efforts to increase opportunities to confront and reflect on biases are needed to avoid perpetuating a prevalent and problematic stigma.

## Abbreviations

(LGBT), Lesbian, Gay, Bisexual, Transgender.

## Competing interests

The authors have no competing interests, financial or otherwise.

## Authors’ contributions

KM designed the study, participated in dissemination and data collection, and drafted the manuscript. RLK and GTM interpreted the data and drafted the manuscript. MDW carried out the statistical analyses and drafted the manuscript. FTF oversaw the study and drafted the manuscript. All authors read and approved the final manuscript.

## Pre-publication history

The pre-publication history for this paper can be accessed here:

http://www.biomedcentral.com/1472-6920/12/71/prepub
